# Targeting the association between telomere length and immuno-cellular bioenergetics in female patients with Major Depressive Disorder

**DOI:** 10.1038/s41598-018-26867-7

**Published:** 2018-06-20

**Authors:** Christina Boeck, Juan Salinas-Manrique, Enrico Calzia, Peter Radermacher, Christine A. F. von Arnim, Detlef E. Dietrich, Iris-Tatjana Kolassa, Alexander Karabatsiakis

**Affiliations:** 1 Clinical & Biological Psychology, Institute of Psychology and Education, Ulm University, Ulm, Germany; 2 AMEOS Klinikum Hildesheim, Hildesheim, Germany; 3 Institute of Anesthesiological Pathophysiology and Process Engineering, University Hospital Ulm, Helmholtzstrasse 8/1, 89081 Ulm, Germany; 4 Department of Neurology, Ulm University, Ulm, Germany; 5 Burghof-Klinik, Rinteln, Germany; 6 Department of Mental Health, Hannover Medical School, Hannover, Germany

## Abstract

Major Depressive Disorder (MDD) has been associated with telomere dysfunction and alterations in mitochondrial activity, which seem to be co-regulated in human cells. To investigate this co-regulation in MDD, we assessed telomere length (TL) in peripheral blood mononuclear cells (PBMC) and selected immune cell subsets by quantitative fluorescence *in situ* hybridization and mitochondrial respiratory activity in PBMC by high-resolution respirometry in a study cohort of 18 MDD patients and 21 non-depressed controls. We provide initial evidence for a differential vulnerability to telomere attrition in selected adaptive immune cell populations. Here we found the highest difference in TL between depressed and control subjects for memory cytotoxic T cells. Depression was associated with reduced mitochondrial activity (mitochondrial bioenergetics), but increased mitochondrial density (mitochondrial biogenesis) in PBMC. Exploratory post-hoc analyses indicated that the changes in TL and immune cell bioenergetics were most pronounced in MDD patients who reported experiences of childhood sexual abuse. Among MDD patients, PBMC TL was as a trend positively associated with mitochondrial density and negatively associated with mitochondrial leak respiration, but not with mitochondrial activity related to biological energy production. These initial findings support the hypothesis of a co-regulation between telomeres and mitochondrial biogenesis but not mitochondrial bioenergetics among MDD patients.

## Introduction

With a lifetime prevalence of 5–12% in men and 10–25% in women^1^, depression is one of the most prevalent mental health disorders and has recently been ranked as the leading cause for mental and physical disability worldwide by the World Health Organization^2^. On a biological level, various alterations in cognitive and somatic functions have already been described in depressed individuals, including profound changes in the nervous, endocrine, and the immune system. Despite this knowledge, the biological entity underlying the pathophysiology of depression still remains elusive. It is, however, a well-established concept that the risk for depression is pivotally influenced by the interaction between individual susceptibility (e.g., genetic predisposition) and the experience of traumatic and stressful life events^3^. Early life adversities such as experiences of abuse, neglect, and maltreatment during childhood thereby emerged as particularly important factors that define the vulnerability to depression in later life. With an increasing number of adverse early life experiences, the life-time prevalence of depression increases in a dose-response relationship^4^. Depression is not only associated with high individual suffering and an increased suicide risk, but also with an increased risk for physical diseases such as cardiovascular disorders, osteoporosis, and neurodegenerative diseases^5,6^, which are generally known as age-related conditions. Accordingly, depression has already been associated with alterations in two major regulators of cellular aging processes: telomeres^7,8^ and mitochondria^9^.

Telomeres, i.e. DNA-protein complexes which cap the chromosomal ends, gradually shorten with each cell division and have therefore been described as biological markers of cellular aging^10,11^. Telomere length (TL) reflects, however, not only the inherent replicative potential of a cell, but is further influenced by psychosocial factors such as lifestyle, physical activity, and chronic or traumatic stress exposure^12^. Two recent meta-analyses consistently concluded that TL is reduced in depressed individuals^8^ and is negatively associated with depressive symptom severity^13^. So far, most results on TL shortening in depression were retrieved from blood leukocytes, which encompass a variety of cell subsets that differ with respect to TL, the rate of TL shortening with age, and *telomerase* activity^14–16^ – an enzyme with the potential to elongate TL that counteracts TL shortening in continuously replicating cells. Only few studies have investigated TL changes in specific immune cell subsets. We previously showed that cytotoxic T cells were more affected by shortened TL than T helper cells and B cells in depressed individuals^7^ and we extended this finding in adult women with childhood maltreatment experiences by showing that the subset of memory cytotoxic T cells was particularly vulnerable to shortened TL in response to psychological stress^17^.

Mitochondria are intracellular compartments of eukaryotic cells and the main producers of biochemical energy in the form of adenosine triphosphate (ATP) by oxidative phosphorylation (OXPHOS) via the mitochondrial respiratory chain. Additionally, mitochondria are also the main production sites of reactive oxygen species (ROS) and therefore critically involved in the regulation of oxidative stress. Whereas the free radical theory of aging has meanwhile been revised – now suggesting that ROS may not be the drivers of biological aging but rather constitute stress signals triggered by age-related damages^18,19^ – alterations in mitochondrial function are still thought to play a major part in the process of cellular aging. In accordance with results from animal models of depression^20^, human studies provided evidence that alterations in mitochondrial dynamics may be involved in the pathophysiology of depression^21,22^. In line with this hypothesis, we showed previously that specific measures of mitochondrial activity, in particular basal oxygen consumption, maximal capacity of the electron transfer system (ETS), respiration related to ATP production, spare respiratory capacity, and mitochondrial coupling efficiency were reduced in immune cells of MDD patients in dependence on the depressive symptom severity^9^. Interestingly, although mitochondrial bioenergetics were reduced in immune cells, we found a significant increase in mitochondrial density suggesting higher levels of mitochondrial biogenesis among depressed individuals.

Just recently, it has been hypothesized that telomeres and mitochondria might be co-regulated in human cells^23–25^. On a biological level, there are several potential pathways which may provide a mutual interaction between telomere and mitochondrial dynamics. First, mitochondria are the main producers of ROS and the telomeric DNA sequence is particularly sensitive to oxidative damages^26^. Second, mitochondria are key regulators of inflammatory processes^27^, whereby chronic low-grade inflammation – a prominent phenotype within depressed patients^28^ – may promote cell turnover and thereby TL attrition in immune cells. Third, there also seems to be a direct link between telomere and mitochondrial dynamics, as it was shown that in telomerase-deficient mice, telomere dysfunction-induced activation of p53 represses the peroxisome proliferator-activated receptor gamma coactivator 1α (PGC-1 α), the master regulator of mitochondrial biogenesis, which might promote mitochondrial dysfunction^23^. Fourth, *telomerase* may constitute another link, as it was shown that under conditions of oxidative stress, ROS production, mtDNA damage, apoptosis and respiratory chain functions are modulated by TERT, the catalytic subunit of the telomere-elongating enzyme^29,30^. In line with these co-regulatory mechanisms, there is a growing body of evidence showing a significant positive association between TL and mitochondrial DNA (mtDNA) copy number in whole blood, both in healthy individuals^31–33^ and individuals with axis I psychopathologies (depression or anxiety disorder) and/or a history of childhood adversity^34^. The whole blood mtDNA copy number was thereby adapted as a measure for cellular mitochondrial density and hence mitochondrial biogenesis, which does, however, not provide any insights into mitochondrial bioenergetics.

Based on this literature knowledge, the aims of the current study were twofold. First, we wanted to investigate whether specific T cell subsets differ in their vulnerability to shortened TL in depressed individuals. Second, we aimed at extending the findings from the literature on the relation between TL and mitochondrial biogenesis to a functional perspective by testing its association with alterations in mitochondrial bioenergetics among depressed and non-depressed individuals.

## Results

### Clinical characteristics of the study cohort

All sociodemographic and clinical variables are summarized in Table 1. Group-wise comparisons between female MDD patients (N = 18) and female, non-depressed control subjects (N = 21) did not reveal any significant differences with respect to age, smoking status, and physical activity. The BMI was, however, significantly higher in the MDD compared to the control group. Depressed individuals showed a significantly higher depressive symptom severity. While 67% of MDD patients were diagnosed with recurrent MDD, 33% were diagnosed with first onset MDD.

**Table 1:** Sociodemographic characteristics and clinical characteristics of depressed patients and non-depressed control subjects.

	Controls	MDD patients	*t/W/χ* ^2a^	*df*	*p*
	(*N* = 21)	(*N* = 18)			
Age (mean ± SD; years)	57.5 ± 5.7	59.3 ± 6.6	−0.92	37	0.37
BMI (mean ± SD; kg/m^2^)	24.5 ± 3.0	29.6 ± 7.5	105.5		**0**.**02**
Smoking status (yes, *N* [%])	3 (14.3%)	8 (44.4%)	2.99	1	0.08
Physical activity (yes, *N* [%])	18 (85.7%)	11 (61.1%)	1.92	1	0.17
BDI-II sum score (mean ± SD)	2.1 ± 2.2	23.8 ± 10.9	−8.33	18.3	**<0**.**001**
Number of traumatic events^b^	1.8 ± 1.9	3.8 ± 3.2	88		**0**.**01**
Childhood sexual abuse (yes, *N* [%])^b^	4 (19.1%)	6 (33.3%)	0.77	1	0.38
*Chronic diseases*					
Hypertension (*N* [%])^c^	4 (19.0%)	4 (22.2%)			
Thyroid disease (*N* [%])^c^	3 (14.3%)	3 (16.7%)			
Arthritis (*N* [%])	2 (9.5%)	1 (5.6%)			
Fibromyalgia (*N* [%])	1 (4.8%)	1 (5.6%)			
COPD (*N* [%])	—	1 (5.6%)			
Asthma (*N* [%])^c^	—	1 (5.6%)			
Esophagitis (*N* [%])^c^	—	1 (5.6%)			
Cardiac disease (*N* [%])	—	1 (5.6%)			
Pain syndrome (*N* [%])	1 (4.8%)	—			
Neurofibromatosis (*N* [%])	1 (4.8%)	—			
Bursitis (*N* [%])	1 (4.8%)	—			
*Medication*					
Antidepressants (*N* [%])	—	13 (72.2%)			
Antipsychotics (*N* [%])^d^	—	5 (27.8%)			
Antihypertensive drugs^e^	3 (14.3%)	7 (38.9%)			
Thyroid hormone (*N* [%])	3 (14.3%)	5 (27.8%)			
Sedatives (*N* [%])	1 (4.8%)	5 (27.8%)			
Analgesics (*N* [%])	—	3 (16.7%)			
Laxatives (*N* [%])	—	2 (11.1%)			
Vitamins (B1,B6,B12) (*N* [%])	—	1 (5.6%)			
Statins (*N* [%])	—	1 (5.6%)			
Blood collection time (mean ± SD)^f^	11.5 ± 2.0	11.0 ± 2.1	0.62	30	0.54

Abbreviations: MDD, Major Depressive Disorder; BDI-II, Beck Depression Inventory II; BMI, Body mass index; SD, standard deviation.

^a^Two-tailed Student’s t-tests/Wilcoxon-Mann-Whitney tests/*χ*^2^ tests. Significant *p*-values are given in bold.

^b^Two missings for MDD patients.

^c^Multimorbidity (three MDD patients, 1 control subject).

^d^Adjunctive treatment to antidepressants.

^e^Antihypertensive drugs included beta blockers, angiotensin receptor blockers, diuretics, calcium channel blockers, and ACE inhibitors.

^f^Blood collection time was calculated as hours from midnight to blood drawings. *N* (Controls) = 18, *N*(MDD patients) = 14.

### Depression-related TL differences in PBMC and T cell subsets

There were no significant group differences in the percentage fractions of T cell subsets between depressed patients and non-depressed control subjects (Table 2). Preparatory analyses revealed across both groups a significant, negative relation between age and TL in naïve T helper cells (*r* = −0.39, *p* = 0.01) and naïve cytotoxic T cells (*r* = −0.42, *p* = 0.008). Age was not significantly associated with TL in PBMC (*τ* = −0.04, *p* = 0.71), memory T helper cells (*r* = −0.16, *p* = 0.34), or memory cytotoxic T cells (*r* = −0.14, *p* = 0.41). TL in PBMC was significantly higher, while TL was significantly reduced in naïve and memory cytotoxic T cells of MDD patients compared to control subjects (Table 2). There were no differences with respect to TL in naïve or memory T helper cells between the two groups. The separate inclusion of age, smoking status, and physical activity as a covariate had thereby no influence on any of the results. Due to the significant group difference between MDD patients and control subjects, the BMI could not be accounted for as covariate in the analyses^35^. We therefore performed subgroup analyses within the group of MDD patients and control subjects to test for an association between TL and BMI. While there were no significant associations between BMI and TL in the MDD group for any of the immune cell types investigated, BMI was negatively correlated with TL in naïve T helper cells among control subjects (*r* = −0.50, *p* = 0.02), but not with TL of any other immune cell subset.

**Table 2:** Immune cell subset composition, telomere length in immune cell subsets and immune cell mitochondrial activity of depressed patients and non-depressed control subjects.

	Controls	MDD	*t/W/χ* ^2a^	*df*	*p*
	(*N* = 21)	(*N* = 18)			
*Immune cell subset composition* (%)^b^					
T cells (CD3^+^)	46.8 ± 13.1	48.8 ± 10.3	−0.52	37	0.61
T helper cells (CD3^+^CD4^+^)	25.4 ± 26.1	26.3 ± 9.1	−0.30	37	0.77
Naïve T helper cells (CD3^+^CD4^+^CD45RA^+^)	14.3 ± 7.2	12.4 ± 7.7	233.0		0.22
Memory T helper cells (CD3^+^CD4^+^CD45RA^−^)	8.8 ± 3.3	10.9 ± 4.8	−1.64	37	0.11
Cytotoxic T cells (CD3^+^CD8^+^)	9.2 ± 3.2	12.2 ± 6.6	140.0		0.17
Naïve cytotoxic T cells (CD3^+^CD8^+^CD45RA^+^)	5.9 ± 2.7	7.9 ± 4.4	−1.77	37	0.09
Memory cytotoxic T cells (CD3^+^CD8^+^CD45RA^−^)	2.2 ± 1.5	3.0 ± 2.5	170.5		0.18
*Telomere length* (*kb*)					
PBMC	7.6 ± 1.1	8.9 ± 1.7	100		**0**.**01**
Naïve T helper cells	7.5 ± 1.4	7.5 ± 1.2	0.04	37	0.97
Memory T helper cells	6.5 ± 1.0	6.0 ± 1.0	1.41	37	0.17
Naïve cytotoxic T cells	7.8 ± 1.6	6.7 ± 1.3	2.34	37	**0**.**03**
Memory cytotoxic T cells^c^	7.1 ± 1.5	5.7 ± 1.1	271		**0**.**008**
*Mitochondrial activity* ^d^					
Routine respiration	4.0 ± 1.1	3.1 ± 1.2	2.48	37	**0**.**02**
Leak respiration^e^	1.4 ± 0.8	1.6 ± 0.6	105.5		0.19
ATP-turnover-related respiration^e^	2.5 ± 0.8	1.3 ± 0.8	4.70	32	**<0**.**0001**
Maximal ETS capacity^e^	8.2 ± 2.6	5.3 ± 1.7	254		**<0**.**0001**
Spare respiratory capacity^e^	4.2 ± 2.2	2.4 ± 1.4	2.90	32	**0**.**007**
ROX^e^	0.5 ± 0.4	0.4 ± 0.3	173.5		0.32
Coupling efficiency (%)^e^	64.5 ± 13.9	41.5 ± 16.1	4.46	32	**<0**.**0001**
Citrate synthase activity^f^ (nmol/min per Mio cells)	0.003 ± 0.002	0.004 ± 0.001	−2.86	35	**0**.**007**

Values are given as mean ± standard deviation.

Abbreviations: MDD, Major Depressive Disorder; CD, Cluster of differentiation; kb, kilobases; ETS, electron transfer system; ATP, adenosine triphosphate; ROX, residual oxygen consumption.

^a^Two-tailed Student’s t-tests or Wilcoxon-Mann-Whitney tests. Significant *p*-values are given in bold.

^b^Percentage of living cells as determined by flow cytometry.

^c^*N* (Controls) = 20 due to insufficient amount of cells.

^d^Given in pmol O2/sec per Mio cells if not stated otherwise.

^e^*N* (Controls) = 18 and *N* (MDD patients) = 16 due to technical problems.

^f^Data of two MDD patients missing due to technical problems; *N* (MDD patients) = 16.

In addition to a group-wise difference, all of the observed TL changes were significantly or marginally significantly associated with depressive symptom severity as assessed by the Becks Depression Inventory (BDI-II) sum score (PBMC: *r* = 0.34, *p* = 0.03; naïve cytotoxic T cells: *r* = −0.31, *p* = 0.06; memory cytotoxic T cells: *r* = −0.50, *p* = 0.001). Furthermore, the BDI-II also showed a significant negative correlation with TL in memory T helper cells (*r* = −0.40, *p* = 0.01).

### Exploratory analyses accounting for the influence of childhood sexual abuse on depression-related changes in immune cell TL

**Figure 1:**
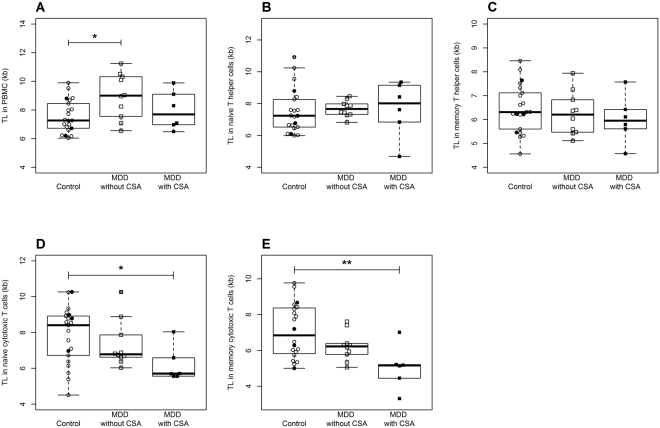
Three-group comparisons of TL in (**A**) PBMC, (**B**) naïve T helper cells, (**C**) memory T helper cells, (**D**) naïve cytotoxic T cells, and (**E**) memory cytotoxic T cells between non-depressed control subjects with (N = 4, filled circles) and without CSA (N = 17, open circles), MDD patients without CSA (N = 12, open squares), and MDD patients with CSA (N = 6, filled squares) revealed a stepwise decrease in TL in naïve and memory cytotoxic T cells, with the shortest TL in MDD patients with CSA. CSA, childhood sexual abuse; kb, kilobase; MDD, Major Depressive Disorder; PBMC, peripheral blood mononuclear cells; TL, telomere length. *p < 0.05, **p < 0.01 (Tuckey post-hoc comparisons).

Adverse childhood experiences such as experiencing childhood sexual abuse (CSA), physical abuse, emotional abuse, and physical or emotional neglect are known to have a long-lasting influence on adult health outcomes. Depressed patients with a history of childhood maltreatment often present with a more severe course of the depressive illness^36^ and are twice as likely to develop chronic and treatment-resistant depression^37^. Given this known influence of adverse childhood experiences on the depressive phenotype, we performed exploratory post-hoc analyses to test whether such differences are also present on a biological level. The MDD group reported significantly more lifetime traumatic events, with 33% reporting to have experienced sexual abuse in childhood compared to 19% in the control group, whereby this group difference did not yield statistical significance. MDD patients with CSA did not differ in any sociodemographic data, lifestyle factors or the current severity of depressive symptoms from MDD patients without CSA (Supplementary Table 1). Four control subjects also reported experiences of sexual abuse during childhood. As these subjects did not differ in any of the sociodemographic or the biological variables of interest (i.e., telomere length and mitochondrial parameters) from controls without CSA, all control individuals were considered as one group in the statistical analyses. As shown in Fig. 1, three-group-comparisons between control subjects, MDD patients without CSA and MDD patients with CSA revealed a significant effect of group on TL in PBMC (*F*[2,34] = 3.98, *p* = 0.03), with the longest PBMC TL in depressed patients without CSA. There were no significant effects on TL in naïve (*F*[2,34] = 0.07, *p* = 0.93) and memory T helper cells (*F*[2,34] = 0.54, *p* = 0.59). Three-group comparisons showed, however, a marginally significant effect of group on TL in naïve cytotoxic T cells (*F*[2,34] = 3.07, *p* = 0.06) and a significant effect on TL in memory cytotoxic T cells (*F*[2,13.7] = 5.68, *p* = 0.02), with a stepwise decrease in TL from non-depressed control subjects over MDD patients without CSA to MDD patients with CSA, who presented the shortest TL.

### Depression-related changes of mitochondrial bioenergetics and biogenesis in immune cells

MDD patients presented with a general decrease in mitochondrial respiratory activity in immune cells and a simultaneous increase in citrate synthase activity indicating a higher mitochondrial density compared to the control group (Table 2). The separate inclusion of age, smoking status, and physical activity as a covariate had no influence on any of the results. Subgroup analyses revealed no significant associations between BMI and mitochondrial variables among MDD patients or non-depressed control subjects.

In addition, all of the observed changes in immune cell mitochondrial activity were significantly correlated with depressive symptom severity (routine respiration: *r* = −0.40, *p* = 0.01; ATP-turnover-related respiration: *r* = −0.62, *p* < 0.0001; maximal ETS capacity: *r* = −0.55, *p* = 0.0009; spare respiratory capacity: *r* = −0.41, *p* = 0.02; coupling efficiency: *r* = −0.59, *p* = 0.0002). Depressive symptom severity also correlated as a trend positively with citrate synthase activity as an estimate for increased cellular mitochondrial density (*r* = 0.28, *p* = 0.09).

### Exploratory analyses accounting for the influence of childhood sexual abuse on depression-related changes in mitochondrial bioenergetics and biogenesis

**Figure 2:**
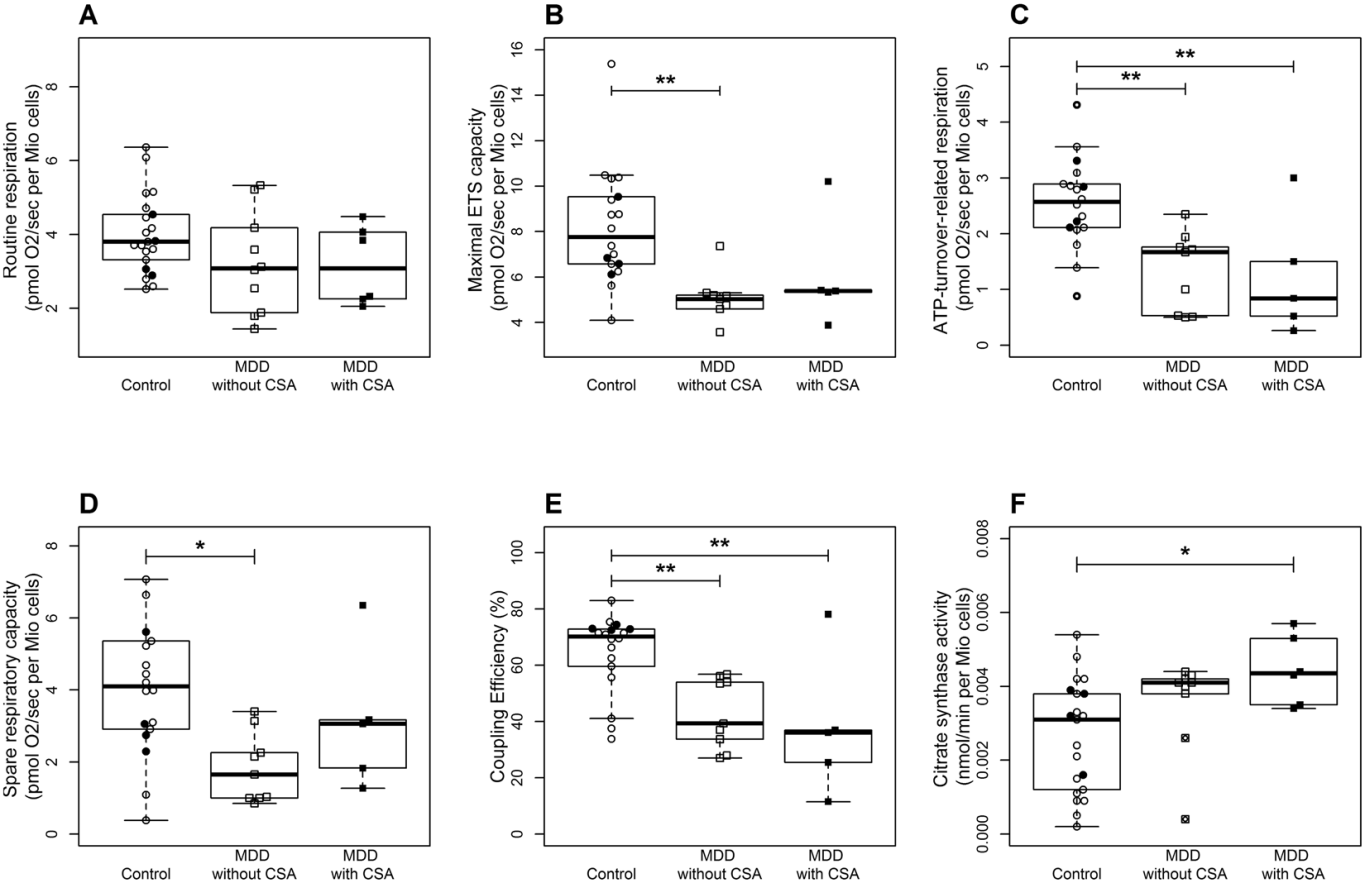
Three-group comparisons of (**A**) mitochondrial routine respiration, (**B**) maximal ETS capacity, (**C**) ATP-turnover-related respiration, (**D**) spare respiratory capacity, (**E**) coupling efficiency, and (**F**) citrate synthase activity between non-depressed control subjects with (N = 4, filled circles) and without CSA (N = 17, open circles), MDD patients without CSA (N = 12, open squares), and MDD patients with CSA (N = 6, filled squares) revealed a stepwise decrease in ATP-turnover-related respiration and coupling efficiency with the strongest reduction in MDD patients with CSA, as well as an increase in the citrate synthase activity, with the highest values for MDD patients with CSA. ATP, adenosine triphosphate; CSA, childhood sexual abuse; ETS, electron transfer system; MDD, Major Depressive Disorder. *p < 0.05, **p < 0.01 (Tuckey post-hoc comparisons).

A three-group-comparison taking the self-reported history of CSA among depressed individuals into account also revealed significant main effects of group on immuno-cellular bioenergetics (Fig. 2): particularly regarding the ATP-turnover-related respiration (*F*[2,31] = 7.82, *p* = 0.002) and the coupling efficiency (*F*[2,31] = 10.92, *p* < 0.001), the analyses showed a stepwise decrease with the highest levels in control subjects with and without CSA, decreased levels in MDD patients without CSA and the strongest decrease in MDD patients with CSA. Additionally, citrate synthase activity was increasing in a stepwise manner (*F*[2,36] = 3.32, *p* = 0.05).

### Associations between TL and immune cell mitochondrial bioenergetics and biogenesis

**Figure 3:**
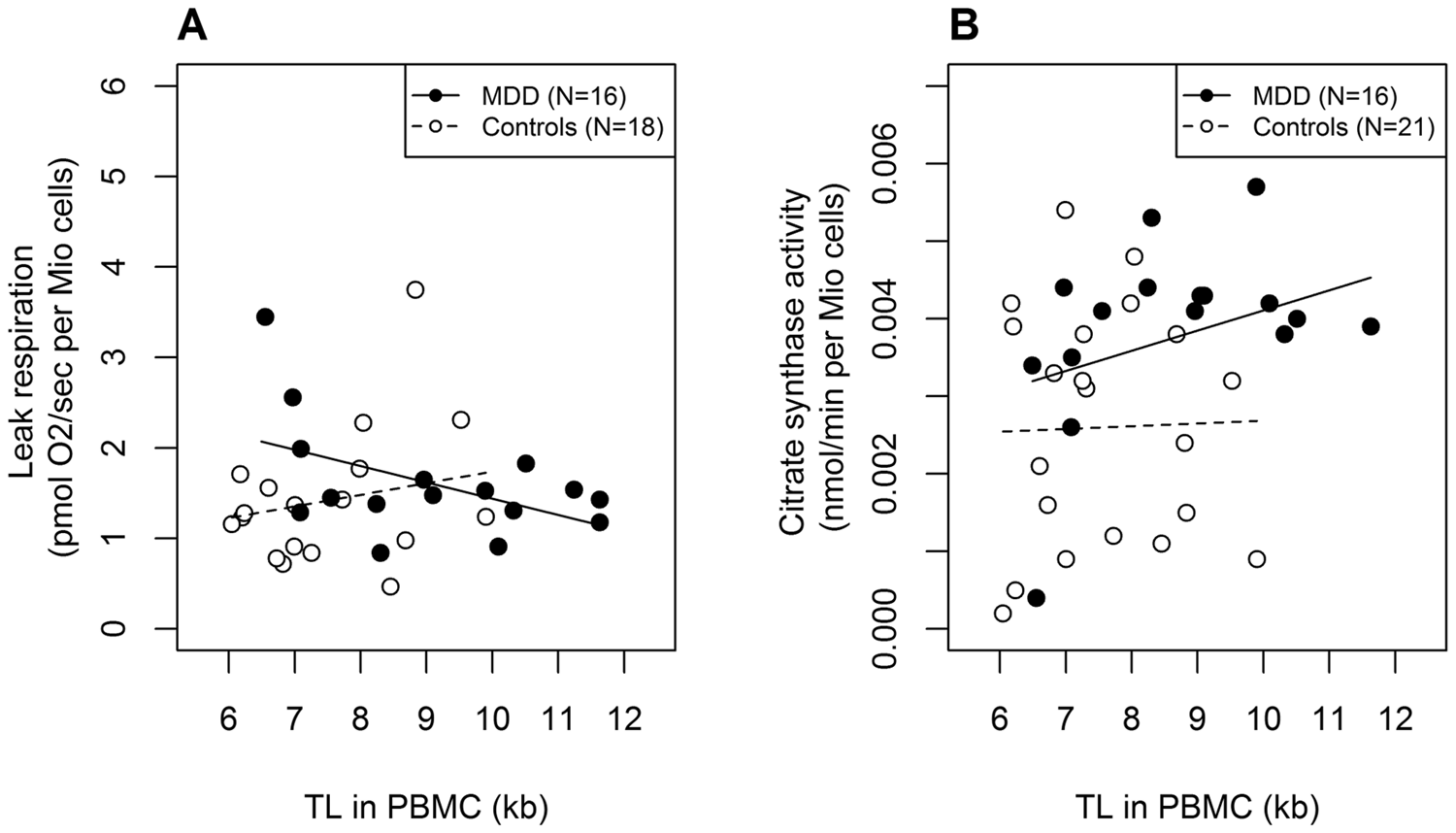
PBMC TL was (**A**) negatively correlated with mitochondrial leak respiration and (**B**) as a trend positively correlated with the citrate synthase activity among MDD patients, but not among non-depressed control subjects. Kb, kilobase; MDD, Major Depressive Disorder; TL, telomere length.

As both mitochondrial activity and TL in PBMC were influenced by group, we performed separate correlation analyses within the subgroups of depressed and non-depressed subjects. PBMC TL was neither associated with mitochondrial activity nor citrate synthase activity in PBMC within the control group (all p-values > 0.05). PBMC TL showed, however, a negative correlation with the leak respiration (*r* = −0.50, *p* = 0.05) and a trend for a positive association with citrate synthase activity (*r* = 0.45, *p* = 0.08) within the group of depressed patients (Fig. 3). There were no further associations between PBMC TL and other mitochondrial measures among MDD patients.

## Discussion

With the present results, we extend the prevailing literature on TL shortening by providing initial evidence for a differential vulnerability of specific T cell subsets for shortened TL in depressed individuals. Consistent with previous findings, TL showed across diverse immune cell subsets a significant association with depressive symptom severity, pointing towards a dose-response effect. Depressed patients presented with reduced mitochondrial activity indicating a decrease in mitochondrial bioenergetics in peripheral immune cells and a simultaneous increase in mitochondrial density indicating higher levels of mitochondrial biogenesis, which was also correlated with the depressive symptom severity. Exploratory post-hoc analyses additionally suggested that a history of adverse childhood experiences in the form of childhood sexual abuse was associated with the biological alterations observed among depressed individuals: MDD patients with a history of CSA showed the most pronounced biological alterations with respect to both, TL shortening and changes in immune cell mitochondrial bioenergetics and biogenesis. While these results clearly need to be replicated in larger studies, this is of particular interest for understanding the inter-individual differences in anti-depressant treatment response and remission rates, which are both worse among depressed patients with a history of childhood maltreatment^36^. Furthermore, we provide evidence for an association between TL and mitochondrial density as well as mitochondrial leak respiration, but no other measures of mitochondrial respiratory activity among MDD patients, whereas TL showed no association with any mitochondrial parameter among non-depressed control subjects.

In contrast to previous studies and the results obtained in isolated T cell subsets, we found an increased TL in the total cell fraction of PBMC of MDD patients compared to age-matched control subjects. There were no changes in the percentage amounts of T cell subsets between depressed and non-depressed individuals. We can therefore exclude that the increase in PBMC TL was driven by a differential subset composition regarding T cell subpopulations, which together constitute 40 to 60% of PBMC. The significant difference in PBMC TL might be attributable to changes within the percentage fractions or TL of B cells, NK cells and/or monocytes. These results further underline the importance to investigate telomere dynamics in distinct cell populations rather than whole blood or PBMC samples.

Regarding T cell subsets, we found that cytotoxic T cells and especially memory cytotoxic T cells were most affected by shortened TL in depressed individuals. This finding is consistent with our previous results from depressed individuals^7^ and adult women with a history of childhood maltreatment^17^ and points towards a profound vulnerability of (memory) cytotoxic T cells for stress-associated biological changes. Which biological characteristics render these cells particularly susceptible for these effects remains to be elucidated in future studies. Potential mechanisms include differences in the lifespan and cycling dynamics of immune cell subsets^38^, which could explain why long-lived memory T cells are particularly affected as they might be most exposed to peripheral stress signals. Additionally, T cell subsets might differ in their responsivity to stress hormones, whereby Choi *et al*. (2008) already showed that cortisol-induced inhibition of telomerase activity was strongest in cytotoxic T cells *in vitro*^39^.

Exploratory post-hoc analyses revealed that the shortened TL in T cell subsets was not only associated with depressive symptom severity, but also most pronounced in MDD patients with CSA. This finding was not attributable to a higher depressive symptom severity in depressed individuals with CSA. It is a replicated finding that MDD patients with a history of childhood maltreatment present a more severe course of the depressive illness^36^ and are twice as likely to develop chronic and treatment-resistant depression^37^. Although MDD patients with and without a history of CSA did not differ in depressive symptom severity and any of the sociodemographic data at the time point of investigation, those with CSA may still have a longer and more complex history of the disease. Thus, individuals who were exposed to stress in early life, might have been exposed to a higher load of cumulative stress exposure over the lifetime, which could account for the observed reduction in immune cell TL. Additionally, biological embedding of early adversities might have reprogrammed stress-sensitive biological trajectories (e.g., by epigenetic modifications of stress-related genes) in a way that renders these individuals more vulnerable for biological changes in response to life stress, thereby promoting adverse health and treatment outcomes in later life^40^.

The same pattern was also found for immune cell bioenergetics, whereby the respiratory activity related to ATP production and the coupling efficiency of the mitochondrial ETS, i.e. the fraction of basal oxygen consumption that is subjected to ATP production, were most reduced in MDD patients with CSA. Simultaneously, citrate synthase activity was highest in these individuals, pointing towards an increased mitochondrial density and hence mitochondrial biogenesis, which is consistent with findings from a previous study^41^. In contrast to the present results, we previously reported that citrate synthase activity was unchanged and mitochondrial activity significantly higher in adult women with childhood maltreatment and without current depressive symptomatology^42^. Alterations in mitochondrial activity therefore seem to be dynamic and might rather constitute state than trait markers following cumulative stress exposure.

In this study, we did not observe any significant differences with respect to the biomarkers under investigation between non-depressed control subjects with and those without a history of childhood sexual abuse. Given the relatively small sample size of control subjects with CSA, these findings should, however, be interpreted with caution. Previous studies already reported an association of adverse early life experiences with immune cell telomere length^17,43,44^ and immune cell mitochondrial activity^42^ among non-depressed individuals. Future studies are needed to further investigate this subject. Within this context, it would also be of high interest to account for potential resilience factors that might buffer the biological consequences of traumatic stress exposure during early life^45^.

In addition to the separate associations of MDD with changes in telomere and mitochondrial dynamics in immune cells, TL in PBMC was negatively associated with leak respiration and as a trend positively associated with citrate synthase activity among depressed individuals. We did not find any significant associations between TL and parameters describing basal mitochondrial respiratory activity or ATP-production-related oxygen consumption, which might indicate that TL is rather associated with mitochondrial biogenesis than with mitochondrial bioenergetics. Several studies consistently reported so far a positive relation between TL and mitochondrial density as assessed by mtDNA copy number in elderly women^31^, pregnant women^32^, and a community sample of healthy women and men^34^. We did not find any significant associations between TL and any of the mitochondrial measures in the control group. Consistent with this finding, Tyrka *et al*. (2015) already reported that the association between TL and mitochondrial density was stronger among subjects who reported both, a history of childhood adversity and a lifetime diagnosis of a psychopathology (*r* = 0.558, *p* < 0.001) than in individuals with no adversity and no psychopathology (*r* = 0.291, *p* = 0.001)^34^.

The present study faces some limitations, including the relatively small sample size, the presence of physical comorbidities, and the concurrent intake of appropriate medications by the study participants, as well as the focus on childhood sexual abuse and the lack of questionnaires to additionally assess other types of adverse early life experiences (physical and emotional abuse or neglect) beside CSA. Additionally, we could not account for the potential influence of BMI on the biological outcomes, as the group of depressed patients and non-depressed control subjects inherently differed with respect to BMI^35^. Rigorous exclusion of depressed patients with a high BMI might, however, have led to the recruitment of a clinically less representative study cohort, as depression is known to be associated with increased BMI among women^46–48^. Within the group of depressed patients and the group of control subjects, we did not see any significant associations between BMI and telomere length or mitochondrial activity, except for a negative relation between BMI and TL in naïve T helper cells among control subjects. These subgroup analyses may suggest that the group differences observed between depressed and non-depressed individuals are not driven by BMI. The here presented findings should, however, be taken as initial results, and need to be replicated in larger study cohorts that can account for the influence of potential confounders such as BMI, comorbidities, medication, or the course of the depressive illness. Furthermore, as we focused on age-progressed women in the present study, the transferability of our findings to younger patients with MDD has to be confirmed in future studies. Finally, to show the generalizability of our findings to men, the same investigations should be replicated in male patients.

Although these limitations have to be kept in mind, this study adds to the accumulating evidence that adverse early life experiences may shape not only clinical, but also biological outcomes among depressed individuals. Additionally, the data support the hypothesis of a co-regulation between telomere and mitochondrial biogenesis in MDD patients, but not mitochondrial bioenergetics. As we provided evidence for a differential vulnerability of specific immune cell subsets, future studies are warranted to investigate cell subset-specific alterations in mitochondrial activity, which might represent a new perspective to unravel the biological mechanisms underlying the immune disturbances and adverse health outcomes observed in depressed individuals.

## Materials and Methods

### Study cohort

Forty-four women within an age range between 50 and 69 years were part of the study, as we hypothesized that changes and group-wise differences in TL would be more pronounced at an advanced age. To exclude known confounding effects of gender-specific differences on TL, only women were included in the study. Depressed patients were recruited during inpatient treatment at the AMEOS Clinic for Psychiatry and Psychotherapy in Hildesheim, Germany. Half of the study cohort consisted of individuals with a current diagnosis of Major Depressive Disorder (MDD) according to DSM-IV^49^. Age-matched controls without any depressive episodes in the past as assessed by self-report were recruited by public poster advertisement (e.g., supermarkets, gyms). The study protocol was in line with the Declaration of Helsinki, approved by the Ethics Committee of the Hannover Medical School and all participants gave written informed consent before participation. Only women without any neuropsychiatric comorbidities including Parkinson’s disease, Alzheimer’s disease and other clinically relevant neurological or psychiatric disorders were included in the study. Furthermore, exclusion criteria comprised the presence of anaemia, severe immune alterations, autoimmune disorders and cancer, as well as the intake of any medication with known effects on the immune system (e.g. immunosuppressors, cytostatic agents, recent vaccination). Additionally, two study participants, one MDD patient and one control subject, showed elevated CRP values (36.3 mg/l and 41.7 mg/l) indicating an acute inflammatory response and were therefore excluded from the analyses.

### Clinical assessments

Both depressed patients and non-depressed control subjects completed the Beck’s Depression Inventory-II (BDI-II, self-report)^50^ for the assessment of current depressive symptom severity. Additionally, the Essener Trauma Inventory (ETI, self-report)^51^ was applied to assess traumatic events across the lifetime and experiences of sexual abuse in childhood (personal experience of “sexual abuse by a family member during childhood or adolescence” or “sexual abuse by a stranger during childhood or adolescence”). Three MDD patients met the criteria for a comorbid diagnosis of PTSD according to the ETI and were therefore excluded from the analyses. To control for confounding demographic variables with known effects on TL, body mass index (BMI), current smoking status, and physical activity (“yes” or “no”) were assessed by self-report at the time point of study participation.

### Isolation of peripheral blood mononuclear cells from whole blood

EDTA-buffered collection tubes (Sarstedt, Nümbrecht, Germany) were used for the collection of whole blood samples by venous puncture between 7 a.m. and 2 p.m. Data on the precise time of blood collection was available from a subcohort of *N* = 32 study participants (*N* = 14 MDD patients and *N* = 18 control subjects). In this subcohort, there was no significant group difference in the time of blood collection between the depressed and the control group (Table 1). Subsequent to blood drawings, peripheral blood mononuclear cells (PBMC) were isolated from whole blood by Ficoll-Hypaque gradient centrifugation according to the manufacturer’s protocol (GE Healthcare, Chalfon St Giles, UK) and isolated cells were stored frozen at −80 °C in cryoprotective freezing medium (dimethyl sulphoxide: Sigma-Aldrich, St. Louis, MO, USA; fetal calf serum: Sigma-Aldrich; dilution 1:10).

### Assessment of mitochondrial respiratory activity in PBMC

Mitochondrial respiratory activity was assessed in intact PBMC by high-resolution respirometry using an O2k-Oxygraph (Oroboros Instruments, Innsbruck, Austria) as described in detail in Karabatsiakis *et al*.^9^. In short, frozen PBMC were thawed, washed with PBS, counted with trypan blue to determine the percentage of viable cells, resuspended in mitochondrial respiration medium Mir05^52^, and 2 ml of cell suspension were each subjected to two chambers of a calibrated O2k-Oxygraph for duplicate measurements and supplemented with 10 µl sodium pyruvate (2 M; Sigma-Aldrich). After basal oxygen consumption (routine respiration) was recorded, the so-called leak respiration (oxygen consumption compensating for cation cycling, proton slippage and proton leak across the inner mitochondrial membrane) and the proportion of oxygen consumption that is used to drive ATP synthesis (ATP-turnover-related respiration; difference between routine and leak respiration) were assessed through addition of the ATP-synthase inhibitor Oligomycin (0.5 µl, 5 mM; Sigma-Aldrich). Subsequently, cells were titrated with FCCP (1 µl, followed by 0.5 µl steps, 1 mM; Sigma-Aldrich) to induce the maximal uncoupled respiration, which is an estimate for the maximal capacity of the mitochondrial electron transfer system (ETS). In six cases, cells did no longer react to FCCP titration following the addition of Oligomycin, therefore only the measures of routine respiration were included in the statistical analyses. To overcome this problem, we exchanged the Oligomycin stock solution used for the measurements, and tested its influence on the subsequent FCCP titration by adding Oligomycin to only one of the two duplicate measurements, which confirmed that it did no longer inhibit the induction of maximal ETS capacity. The spare respiratory capacity was calculated by subtracting the routine respiration from the maximal ETS capacity. At last, the complex I inhibitor Rotenone (5 µl, 1 mM; Sigma-Aldrich) and the complex III inhibitor Antimycin A (1 µl, 5 mM; Sigma-Aldrich) were added to block the electron transfer along the mitochondrial ETS in order to measure the proportion of oxygen consumption that is attributable to other cellular processes than the OXPHOS system (residual oxygen consumption, ROX). All primary values were corrected for ROX. Furthermore, the coupling efficiency (ATP-turnover-related respiration over uncoupled respiration) was calculated for further interpretation of changes in mitochondrial activity.

Citrate synthase activity, a well-established and robust marker for the mitochondrial density per cell^53^, was assessed in shock-frozen PBMC by spectrophotometry as described previously^54^. Averages of duplicate measurements were used for statistical analyses. Due to technical problems in the measurement of citrate synthase activity, the values of two MDD patients are missing.

### Isolation of T cell subsets

Following respirometry, cell suspensions were centrifuged and PBMC were resuspended in cell separation buffer (PBS, 0.5% bovine serum albumin, 2 mM EDTA; Miltenyi Biotec, Bergisch-Gladbach, Germany) for the separation of T cell subsets. Appropriate volumes of antibodies (CD4-APC [10 µl/10^7^ cells], CD8-FITC [10 µl/10^7^ cells], CD45RA-PE [5 µl/10^7^ cells], CD3-PE-Vio770 [5 µl/10^7^ cells], all purchased from Miltenyi Biotec) were added and cells were stained according to the manufacturer’s protocol. Naïve T helper cells (CD3^+^CD4^+^CD45RA^+^), memory T helper cells (CD3^+^CD4^+^CD45RA^−^), naïve cytotoxic T cells (CD3^+^CD8^+^CD45RA^+^), and memory cytotoxic T cells (CD3^+^CD8^+^CD45RA^−^) were then isolated by fluorescent-activated cell sorting on a BD FACSAria III cell sorter (BD Biosciences, Heidelberg, Germany). Propidium iodide staining was used to distinguish dead from living cells.

### Telomere length analysis

Following isolation, cells were fixated in a 3:1 solution of methanol (Sigma-Aldrich) and glacial acetic acid (VWR, Radnor, PA, USA) and subjected to quantitative fluorescence *in situ* hybridization (qFISH) for TL assessment as described previously^17^. In short, 10^5^ cells were spread out on *superfrost* slides (Menzel-Glaeser, Braunschweig, Germany) and washed for 5 min in PBS. Subsequently, cells were permeabilized through incubation in a pepsin solution for 10 min at 37 °C, washed in PBS (2 × 5 min) and stained for 2 hours at room temperature with Cy3-labeled peptide nucleic acid telomere oligonucleotides (Panagene, Daejeon, Korea) in a humid chamber. Following two washing steps (20 min) with washing buffer (formamide, 1 M Tris and 10%-BSA), one washing step with TBS-Tween (1%) (5 min), and two washing steps with PBS (5 min), cell nuclei were counterstained with DAPI (Vectashield Mounting Medium; Vector Laboratories, Burlingame, CA, USA).

Analysis of stained cells was performed using a digital monochromatic AxioCamMRm camera (Carl-Zeiss-Microscopy, Jena, Germany) mounted on an AxioVert200 inverted fluorescent microscope (Carl-Zeiss-Microscopy, Germany). Images were captured at a 1000-fold magnification at standardized settings concerning gain and exposure time. The telomere fluorescence intensity (TFI), which correlates with the respective TL, was assessed in 100 cells per sample with the image acquisition software TFL-TeloV2^55^ and mean TFI was calculated as an average of all TFI values per sample.

### Standard curve generation for conversion of TFI to kilobases

To estimate the corresponding kilobase (kb) value of mean TFI, a standard curve was generated consisting of six cell lines (BJ, HeLa, HeLa(YFP)hTERT, HeLa(YFP)hTERT TERC, HuH7, IMR90). DNA of cultured cells was extracted using the DNeasy Blood and Tissue Kit (Qiagen, Venlo, Netherlands) and subjected to southern blot analysis for determination of telomere restriction fragment (TRF) length. In short, duplicates of DNA samples (5 µg) were digested overnight at 37 °C with restriction enzymes *HinfI* and *RsaI* (New England Biolabs, Ipswich, MA, USA), complete digestion was confirmed by gel-electrophoresis and restriction fragments and a DNA ladder (1Kb plus DNA Ladder, Invitrogen, Carlsbad, CA, USA) were separated by electrophoresis on 0.7% agarose gels. The gel was vacuum-dried for 1 hour at 60 °C, denatured in 1.5 M NaCl/0.5 M NaOH and neutralized in 1 M Tris/1.5 M NaCl. 5′^32^P-labeled telomere oligonucleotides were used for in-gel hybridization (overnight at 37 °C). The gel was washed three times in 0.1 × SSC/0.1% SDS at 37 °C (15 min/wash) and analysed using the fluorescent image analyser FLA2000 system (FujiFilm Imaging System, Willich, Germany). The PCBAS Southern Blot software Version 2.0 (Raytest Isotopenmessgeräte GmbH, Germany) was used to assess TRF length quantitatively over the whole lane. Based on the DNA migration distance, mean TRF length was determined by comparing the background-corrected average in optical density with the 1Kb plus DNA Ladder.

The six standard cell lines were co-stained with each qFISH run. Based on the TRF length assessed by southern blot analysis and the respective TFI values determined for the six cell lines, a linear regression model was administered to generate a standard curve for each individual experiment (*R*^2^ = 0.85–0.95). This approach allowed to control for inter-assay variability and to estimate the kb values corresponding to TFI values assessed by qFISH.

### Statistical analysis

Statistical analyses were performed with R version 3.3.2^56^. Normal distribution of residuals was determined by Shapiro Wilk tests and non-parametric analyses were chosen where appropriate. Group comparisons between the depressed and the control group regarding sociodemographic, clinical, and biological variables were calculated by Chi-squared tests for categorical variables and two-tailed Student’s *t*-tests (parametric) or Wilcoxon-Mann-Whitney tests (non-parametric) for continuous variables. To account for the influence of potential confounders (age, smoking status, physical activity, BMI) on TL and immune cell bioenergetics, linear regression analyses were performed in a second step. Two-tailed Pearson’s correlation coefficients (parametric) and Kendall’s *τ*-correlation coefficients (non-parametric) were calculated for correlation analyses. To investigate the influence of childhood sexual abuse (CSA), study participants were divided into subgroups of individuals with and without CSA and compared by ANOVA analyses (Tuckey post-hoc comparisons).

### Data availability

In accordance with the informed consent given by the study participants, the anonymized datasets generated during and/or analysed during the current study are available from the corresponding author on reasonable request.

## Electronic supplementary material

Supplementary Table 1
