# Supplementary material for: Targeting the association between telomere length and immuno-cellular bioenergetics in female patients with Major Depressive Disorder
Source: Sci Rep. 2018 Jun 20;8:9419. doi: 10.1038/s41598-018-26867-7 (PMC6010455; doi:10.1038/s41598-018-26867-7)
Supplement: Supplementary file 1 — Supplementary Table 1 [file 41598_2018_26867_MOESM1_ESM.docx]

***Supplementary Information***

**Targeting the association between telomere length and immuno-cellular bioenergetics in female patients with Major Depressive Disorder**

Boeck, Christina *^1^, Juan Salinas-Manrique ^2^, Enrico Calzia ^3^, Peter Radermacher ^3^, Christine A.F. von Arnim ^4^, Dietrich, Detlef E. ^5,6^, Kolassa, Iris-Tatjana ^1^, Karabatsiakis, Alexander ^1^

^1^ Clinical & Biological Psychology, Institute of Psychology and Education, Ulm University,

Ulm, Germany

^2^ AMEOS Klinikum Hildesheim, Hildesheim, Germany.

^3^ Institute of Anesthesiological Pathophysiology and Process Engineering, University Hospital Ulm, Helmholtzstrasse 8/1, 89081 Ulm, Germany.

^4^ Department of Neurology, Ulm University, Ulm, Germany.

^5^ Burghof-Klinik, Rinteln, Germany

^6^ Department of Mental Health, Hannover Medical School, Hannover, Germany

*Corresponding author: Christina Boeck, Clinical & Biological Psychology, Institute of

Psychology and Education, Ulm University, Albert-Einstein-Allee 47, 89081 Ulm, Germany.

Telephone: 0049 731 5026596. Fax: 0049 731 5026599. Mail: christina.boeck@alumni.uni-ulm.de and alexander.karabatsiakis@uni-ulm.de

| Supplementary Table 1. Sociodemographic characteristics and clinical characteristics of depressed patients with and without a history of childhood sexual abuse ^1^. | | | | | | |
| --- | --- | --- | --- | --- | --- | --- |
|  | | MDD patients  with CSA | MDD patients  without CSA | *t/W/χ^2^* ^2^ | *df* | *p* |
|  |  | (*N* = 6) | (*N* = 10) |  |  |  |
| Age (mean ± SD; years) | | 61.3 ± 7.9 | 57.4 ± 6.2 | -1.11 | 14 | 0.29 |
| BMI (mean ± SD; kg/m^2^) | | 28.8 ± 6.1 | 29.6 ± 7.0 | 33 |  | 0.79 |
| Smoking status (yes, *N* [%]) | | 3 (50.0 %) | 4 (40.0 %) | 0.15 | 1 | 0.70 |
| Physical activity (yes, *N* [%]) | | 4 (66.7 %) | 5 (50.0 %) | 0.20 | 1 | 0.90 |
| BDI-II sum score (mean ± SD) | | 19.5 ± 12.9 | 27.4 ± 9.8 | 1.39 | 14 | 0.19 |
| Recurrent MDD (yes, *N* [%]) | | 5 (83.3 %) | 6 (60.0 %) | 0.95 | 1 | 0.33 |
| Number of traumatic events | | 5.8 ± 4.2 | 2.5 ± 1.7 | 10.5 |  | **0.04** |
| *Chronic diseases* | |  |  |  |  |  |
|  | Hypertension (*N* [%]) | 1 (16.7 %) | 3 (30.0 %) |  |  |  |
|  | Thyroid disease (*N* [%]) | 2 (33.3 %) | 1 (10.0 %) |  |  |  |
|  | Fibromyalgia (*N* [%]) | - | 1 (10.0 %) |  |  |  |
|  | COPD (*N* [%]) | 1 (16.7 %) | - |  |  |  |
| *Medication* | |  |  |  |  |  |
|  | Antidepressants (*N* [%]) | 6 (100.0 %) | 5 (50.0 %) |  |  |  |
|  | Antipsychotics (*N* [%]) ^3^ | 3 (50.0 %) | 1 (10.0 %) |  |  |  |
|  | Antihypertensive drugs ^4^ | 5 (83.3 %) | 4 (40.0 %) |  |  |  |
|  | Thyroid hormone (*N* [%]) | 4 (66.7 %) | 1 (10.0 %) |  |  |  |
|  | Sedatives (*N* [%]) | 3 (50.0 %) | 2 (20.0 %) |  |  |  |
|  | Analgesics (*N* [%]) | 1 (16.7 %) | 2 (20.0 %) |  |  |  |
|  | Laxatives (*N* [%]) | 1 (16.7 %) | 1 (10.0 %) |  |  |  |
|  | Vitamins (B1,B6,B12) (*N* [%]) | 1 (16.7 %) | - |  |  |  |
|  | Statins (*N* [%]) | 1 (16.7 %) | - |  |  |  |
| Abbreviations: CSA, childhood sexual abuse; MDD, Major Depressive Disorder; BDI-II, Beck Depression Inventory II; BMI, Body mass index; SD, standard deviation. | | | | | | |
| 1: Data on the Essener Trauma Inventory (ETI) and thus childhood sexual abuse was missing for two MDD patients, who were excluded for all analyses regarding a history of CSA. | | | | | | |
| 2: Two-tailed Student’s t-tests/Wilcoxon-Mann-Whitney tests/*χ*2 tests. Significant *p*-values are given in bold. | | | | | | |
| 3: Adjunctive treatment to antidepressants.  4: Antihypertensive drugs included beta blockers, angiotensin receptor blockers, diuretics, calcium channel blockers, and ACE inhibitors. | | | | | | |
